# Blocking the Mineralocorticoid Receptor Improves Cognitive Impairment after Anesthesia/Splenectomy in Rats

**DOI:** 10.7150/ijms.48767

**Published:** 2021-01-01

**Authors:** Xixia Feng, Lu Chen, Ruihao Zhou, Xiuqun Bao, Hongxia Mou, Ling Ye, Pingliang Yang

**Affiliations:** 1 Department of Pain Management, West China Hospital, Sichuan University, Chengdu, Sichuan Province, 610041, P. R. China.; 2 Department of Anesthesiology, The First Affiliated Hospital of Chengdu Medical College, Xindu, Sichuan, 610500, P. R. China.

**Keywords:** Mineralocorticoid receptor, Cognitive decline, Postoperative cognitive dysfunction, Eplerenone, Neuroinflammation

## Abstract

Recent mounting studies showed that neuroinflammation caused by surgery or anesthesia is closely related to postoperative cognitive dysfunction (POCD). This study investigated the effect of mineralocorticoid receptor (MR) on neuroinflammation and POCD. To detect the MR effect in an animal model, we randomly divided rats into control, anesthesia, and surgery groups. To determine whether the MR-specific blocker eplerenone (EPL) could improve cognitive dysfunction, we assigned other animals into the control, surgery and EPL treatment, and surgery groups. Cognitive function was detected using the Morris water maze. Serum cytokine levels were measured by ELISA, and the histopathological changes of hippocampal neurons were identified by hematoxylin/eosin and Nissl staining. Our research confirmed that anesthesia and surgical stimulation could lead to IL-1β, IL-6, and TNF-α activation and hippocampal neuronal degeneration and pathological damage. MR was upregulated in the hippocampus under cognitive impairment condition. Additionally, EPL could alleviate inflammatory activation and neuronal damage by exerting neuroprotective effects. The preclinical model of sevoflurane anesthesia/splenectomy implied that MR expression is upregulated by regulating the neuroinflammation in the brain under POCD condition. Manipulating the MR expression by EPL could improve the inflammation activation and neuronal damage.

## Introduction

Postoperative cognitive dysfunction (POCD) is a common complication of the central nervous system (CNS) after anesthesia and surgery, mainly affecting the memory, learning, orientation, cognitive, and executive abilities of patients with normal cognitive function before surgery 1, 2. POCD is also associated with prolonged hospital stays, delayed rehabilitation, and increased mortality 3. Therefore, exploring the mechanism of POCD and identifying prevention targets are crucial. However, POCD mechanism remains poorly understood, and no precise treatment strategy has been proposed.

Neuroinflammation caused by surgery or anesthesia is closely related to POCD 4. Regardless of the types of surgery, patients with POCD experience an increase in proinflammatory cytokine expression in serum and cerebrospinal fluid 5, 6. Consistently, studies on various animal surgical models have shown that proinflammatory cytokines and inflammatory mediators are upregulated in peripheral tissues and in the CNS with increased POCD incidence 4, 7, 8. Proinflammatory cytokines can adversely affect the regulation of hippocampal neurotransmitter signals, causing neuronal damage and cognitive dysfunction 9, 10. Considerably numerous cytokine receptors are found in the hippocampus, making it susceptible to high concentrations of inflammatory cytokines such as IL-1β and TNF-α during neuroinflammation 10.

Mineralocorticoid receptor (MR) is a member of the nuclear receptor superfamily that expresses limitedly in the brain; it mainly exists in the hippocampus, lateral nucleus, amygdala, part of the brain stem, and the hypothalamus medium 11. Additionally, MR is involved in the aldosterone- or glucocorticoid-controlled processes, such as cardiovascular homeostasis, adipocyte differentiation or neurogenesis, and neuronal activity regulation in the hippocampus 12, 13. Surgery-induced stress and trauma contribute to the activation of the hypothalamic-pituitary-adrenal (HPA) axis and the release of the stress hormone cortisol, which acts via glucocorticoid receptors (GR), and the MR in the brain could influence various cognitive functions 14. Importantly, patients with POCD undergoing hip fracture surgery have prominently higher cortisol levels than those with non-POCD 15. Our previous studies 16-18 discovered that MR is activated by proinflammatory reaction stimulation, tissue damage, and pain behavior in the local inflammation of lumbar dorsal root ganglia (DRG), representing the low back pain model. In addition, the highly selective MR-specific blocker eplerenone (EPL) could alleviate mechanical pain and excessive sensory neuron excitability in animals. Gabriela M et al. 19 found that EPL could significantly inhibit the cardiomyocyte ICAM-1 expression and macrophage infiltration. However, the stimulation of MR and its contribution to the neuroinflammation response in POCD development remain inadequately investigated. Thus, exploring the effect of MR on POCD development is of great interest.

Hence, this study aimed to investigate whether or not MR is upregulated under surgical conditions and EPL could improve the cognitive dysfunction in rats by inhibiting the neuronal inflammatory response during POCD development.

## Materials and methods

### Animals and experimental design

This study used male Sprague Dawley rats (aged 2 months, 200-250 g), which were purchased from Chengdu DaShuo Experimental Animal Co., Ltd. in Chengdu, China. All experiments conformed to the guidelines published by the Ethics Committee for Animal Experiments of Chengdu Medical College (in accordance with the standards formulated by the national ministerial Experimental Animal Management Committee). All rats were housed under controlled conditions on the ambient temperature of 23°C ± 2°C, relative humidity of 50%, sufficient water and food, and a 12 h light/dark cycle. To eliminate the influence of environmental discomfort in rats, we began the experiment 7 days later.

**Figure 1:**
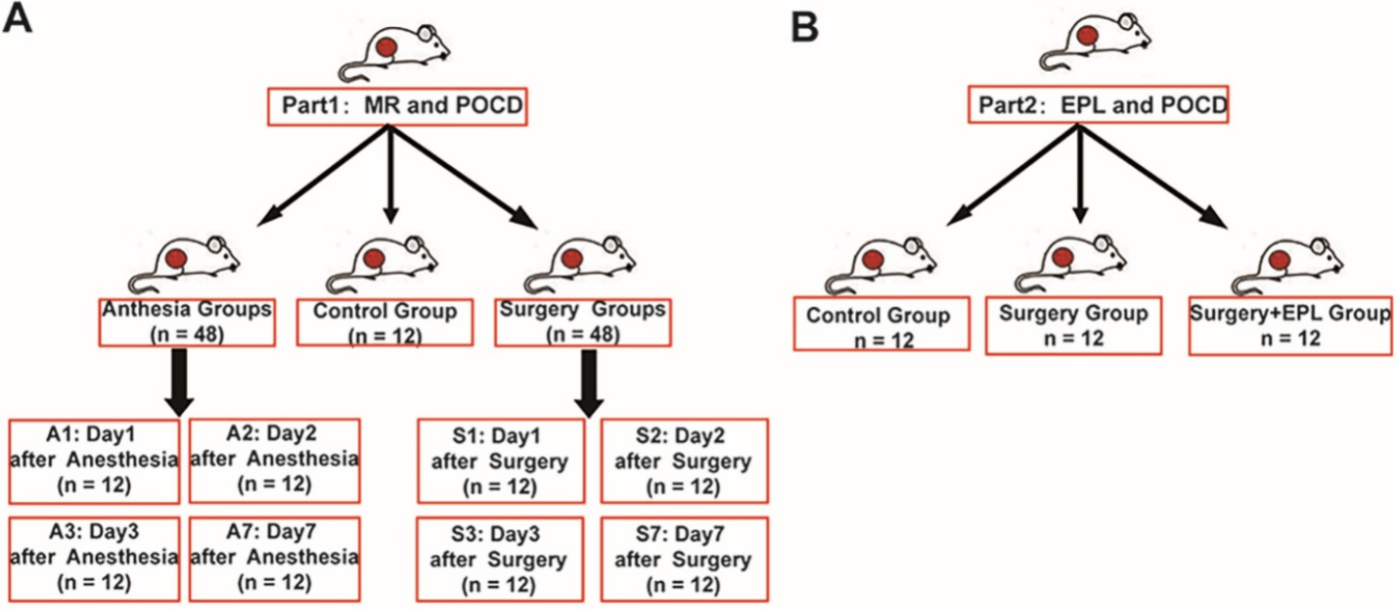
** Experimental design and grouping.** (A) To explore the role of MR in POCD, we divided 108 animals into groups: control group (C, n = 12), anesthesia group (A1, A2, A3, A7, n = 12/group), and surgery group (S1, S2, S3, S7, n = 12/group). (B) To explore the role of EPL in POCD, we divide them into Control group (C, n = 12), Surgery and EPL treatment group (S+E, n = 12), and Surgery group (S, treatment with the same amount of normal saline, n = 12).

To explore the role of MR in POCD, we divided 108 rats into groups: control group (C, n = 12), anesthesia group (A1, A2, A3, A7, n = 12/group, 1, 2, 3, 7 represented the day after the anesthesia), and surgery group (S1, S2, S3, S7, n = 12/group, 1, 2, 3, 7 represented the day after the surgery) (Figure 1A). Moreover, to detect whether EPL could improve the cognitive dysfunction, we randomly assigned other rats to three groups: Control group (C, n = 12), Surgery and EPL treatment group (S+E, n = 12), Surgery group (S, treatment with the same amount of normal saline, n = 12) (Figure 1B).

### Animals and drug treatment

The experiment was started 7 days later. Preoperatively, the rats in each group fasted for 12 hours and drank freely until 2 hours. As previously reported 20, we established the animal model of POCD via anesthesia and splenectomy. The anesthesia group was induced by 3% sevoflurane inhalation, analgesia with buprenorphine (0.08 mg/kg s.c.) and maintained with 2% sevoflurane for 2 hours. The surgery and anesthesia groups underwent the same anesthesia conditions, whereas the control group did not receive any interventions. Splenectomy was simply a small incision in the left upper quadrant to expose, separate and remove the spleen. The wound was infiltrated with 0.25% bupivacaine and sutured. This procedure was chosen because it represented a standardized organ or tissue removal intervention and a moderate surgical procedure.

The effect of EPL was tested as follows: the Surgery and EPL treatment group received EPL (100 mg/kg) by gavage 30 minutes preoperatively, whereas the Surgery group received normal saline (100 mg/kg) 30 minutes preoperatively. The surgeries of both groups were performed as previously mentioned 20.

### Detection of cognitive function: behavior test

As previously described 21, 22, rat behavior was tested for hippocampal-dependent memory and cognitive function by using the Morris water maze (MWM). The MWM test was mainly included in three processes, namely, acquisition phase, probe trial phase, and reversal phase. In acquisition phase, the rats were placed in the water maze for 5 continuous days preoperatively. We randomly placed them into the water from the pool wall of four quadrants 4 times individually and then measured the escape latency by recording the time from entering the water to boarding the platform. On the 5th day, we anesthetized or surgically treated them per group. In probe trial phase, we removed the original platform and released the rats in the water from the opposite side of the original platform quadrant on days 1, 2, 3, and 7 after treatment. In reversal phase, right after the robe trial phase, the immediate working memory ability was determined by using the working memory test, which was performed by observing whether the rats could learn quickly and memorize the new goal position. Escape latency, swimming speed, frequency of crossing the platform and the percentage of staying time in target quadrant were recorded as the main detection index.

### Determination of serum cytokine levels

At the end of the behavior test, we anesthetized the rats and fully exposed their hearts. Then, we centrifuged the whole blood from the right atrium for 20 minutes and refrigerated the upper serum at -80°C. The serum IL-1β, IL-6, and TNF-α levels were measured via a commercial ELISA kit (BD Biosciences Co., San Jose, CA, USA) according to the manufacturer's protocol.

### Brain tissue preparation and pathological examination

After the behavioral test, the brain tissue of the rats in each group was removed, stored in 4% paraformaldehyde, and fixed at 4°C for 48 hours. We embedded the samples in the paraffin and sliced them into 5 µm-thick sections. After deparaffinization and rehydration, the tissues were stained with hematoxylin/eosin (HE) and Nissl to detect the histopathological changes of hippocampal neurons under light microscope.

### Immunohistochemistry

Briefly, the paraffin section was dewaxed and hydrated in xylene. Thereafter, the samples were blocked in 2% normal goat serum and incubated with MR-specific antibody (1:100, cat: GR195021-12, Abcam) overnight at 4°C. The slides were probed with Biotin-labeled Goat anti-rabbit IgG (PV6001; OriGene Technologies, Inc., Beijing, China) for 30 minutes.

### Western blot

The hippocampal tissue of rats in each group was separated and sliced into pieces. We centrifuged the hippocampal tissue homogenate and extracted protein according to the kit instructions. Furthermore, we quantified the amounts of MR, TNF-α, IL-6, and IL-1β expression in hippocampal neurons by Western blot analysis.

### Statistical methods

All statistical data were analyzed using the SPSS 20.0 software, and the measurement data were presented as mean ± standard deviation (x ± SD). The data among groups were analyzed by repeated measures analysis of variance. Moreover, two groups were compared using the LSD method. Significance was ascribed for *P* < 0.05. The level of significance is determined by the number of symbols, for example, *P = 0.01 to <0.05; **P = 0.001 to 0.01; ***P < 0.001.

## Results

### Anesthesia and splenectomy induced cognitive impairments in rats

**Figure 2:**
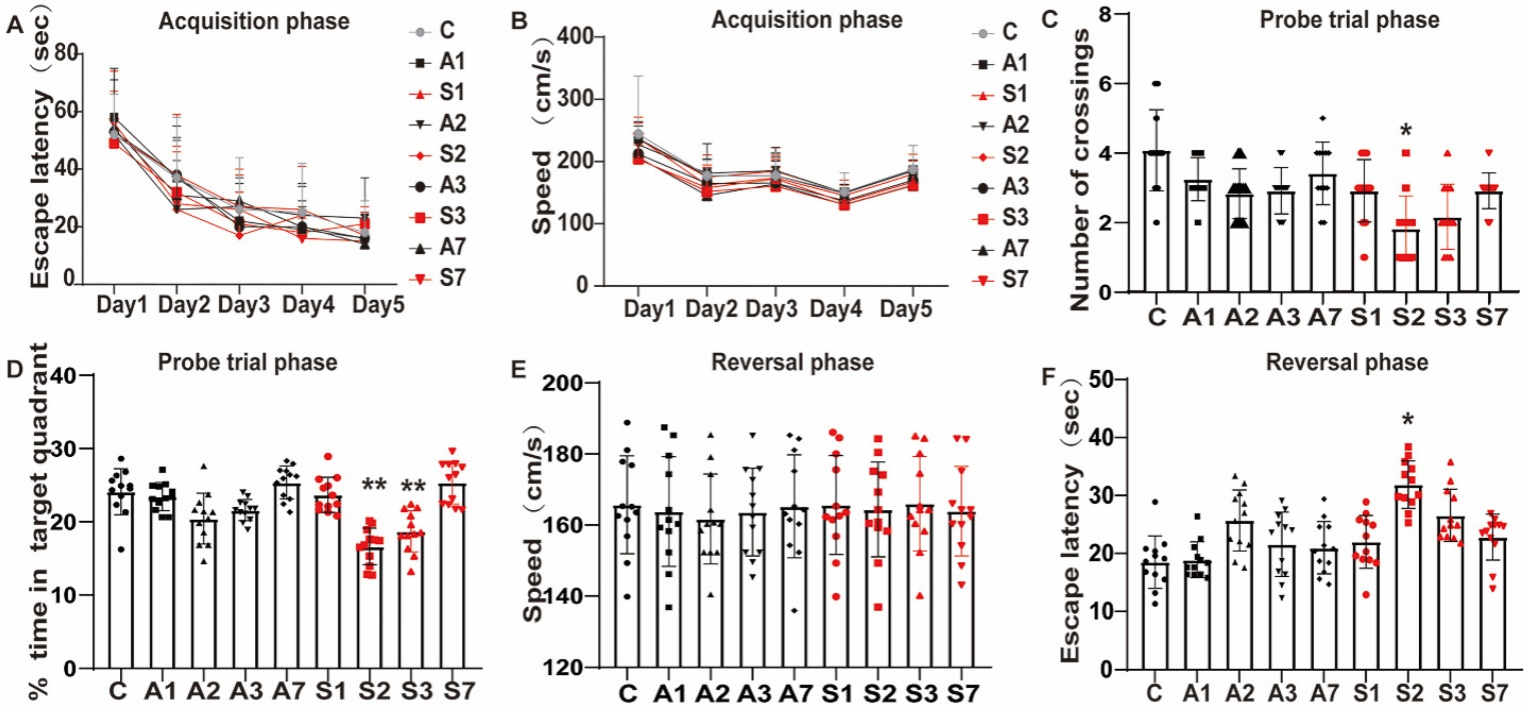
** Cognitive function impairment after anesthesia and splenectomy in rats.** (A) Escape latency and (B) swimming speed in the acquisition phase. Anesthesia and surgery caused cognitive impairment during the probe trial and reversal phases on days 1, 2, 3, and 7 after treatment. (C) Frequency of crossing the platform and (D) the time percentage of staying in the target quadrant during the probe trial phase. (E) Swimming speed and (F) escape latency in the reversal phase. Data are presented as the mean ± SD. *P < 0.05 and **P < 0.01 vs. the control group. Control group (C, n = 12); Anesthesia groups after days 1, 2, 3, and 7 (A1, A2, A3, and A7, n = 12); Surgery groups after days 1, 2, 3, and 7 (S1, S2, S3, and S7, n = 12).

To evaluate the effect of the anesthesia and surgery trauma on the learning and memory abilities of rats, we detected their spatial learning and memory abilities by using the MWM. From day 5 before the treatment, the escape latency in each group gradually improved over time during the acquisition phase (*P* < 0.05, Figure 2A), indicating that animals formed a spatial memory for the surrounding environment through training. Additionally, no significant difference was observed in escape latency and swimming speed throughout the groups at the same time point (*P* > 0.05, Figure 2A-B).

At days 1, 2, 3, and 7 after the treatment, probe trial test and reversal test were conducted. The frequency of crossing the platform was significantly reduced in the S2 group (*P* < 0.05, Figure 2C), as well as the time percentage in the S2 (*P* < 0.01, Figure 2D) and S3 (*P* < 0.01, Figure 2D) groups in the probe trial phase. Likewise, the escape latency of reversal test was significantly longer in the S2 group (*P* < 0.05, Figure 2F). No significant difference was found between the Control and the day 1 and 7 groups (*P* > 0.05), but a trend of cognitive impairment was observed. No changes were reported in swimming speed during the acquisition and reversal phase across the groups, suggesting that anesthesia and surgery did not affect the motor ability of animals (Figure 2B and E). The data revealed that the stimulation of anesthesia and splenectomy might lead to cognitive function decline, which appeared on day 1 up to days 2 and 3 and gradually recovered on day 7. Notably, splenectomy induced a significant cognitive impairment.

### Increased serum level of inflammatory cytokines and damaged hippocampal neurons under cognitive impairment condition

**Figure 3:**
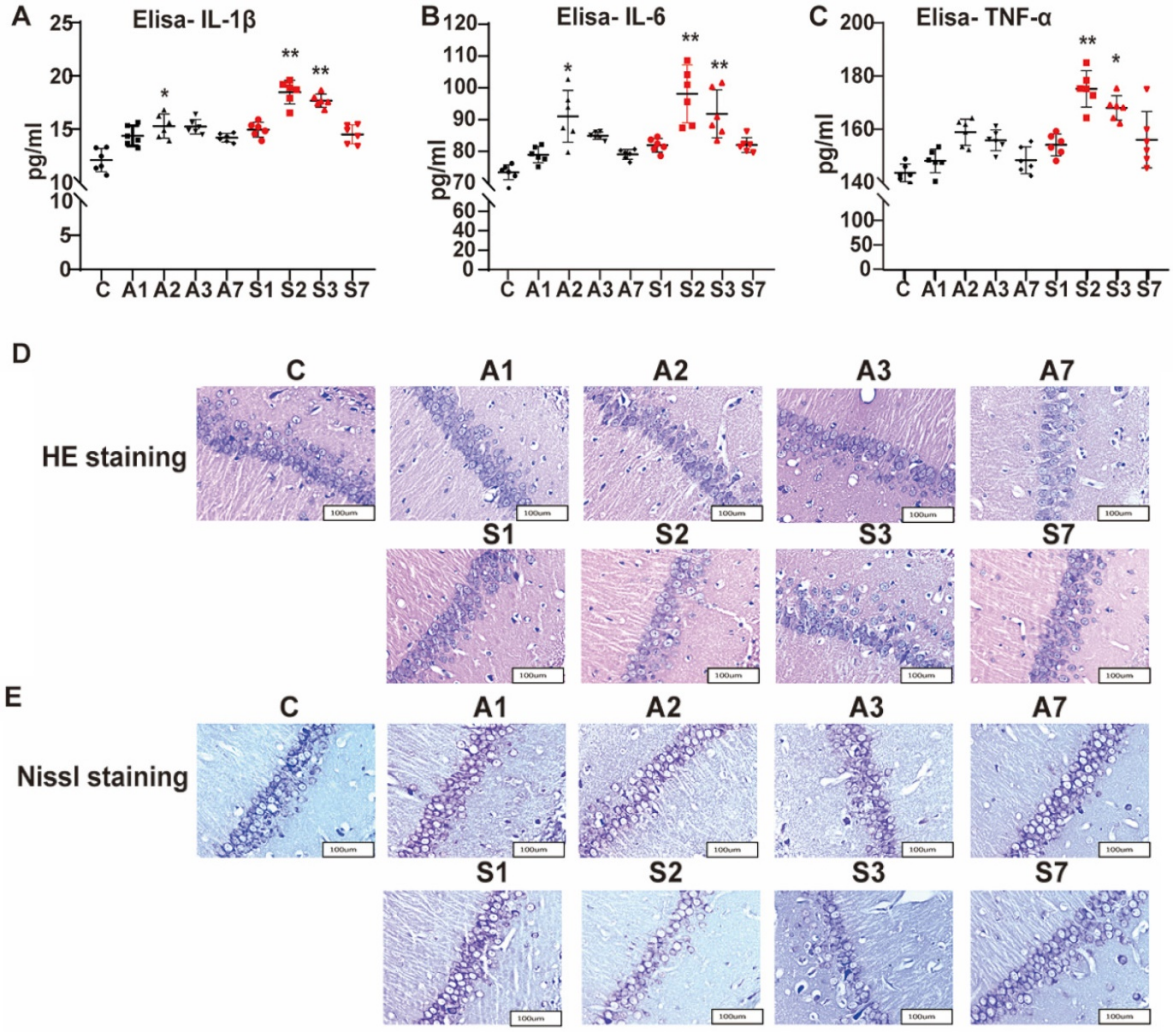
** Increased serum level of inflammatory cytokines and damaged hippocampal neurons under cognitive impairment condition.** The expression of serum inflammatory cytokines (A) IL-1β, (B) IL-6, and (C) TNF-α among the groups was assayed using ELISA. (D) HE and (E) Nissl staining in the hippocampal CA1 region to detect the histopathological changes of hippocampal neurons among the groups. IL, interleukin; TNF, tumor necrosis factor; HE, hematoxylin/eosin. Data are presented as the mean ± SD. *P < 0.05 and **P < 0.01 vs. the control group. Control group, n = 6; A1, A2, A3, and A7 groups, n = 6; S1, S2, S3, and S7 groups, n = 6.

After the behavioral test, we detected the serum levels of IL-1β, IL-6, and TNF-α from the peripheral inflammation aspect by using ELISA. Compared with the control group, IL-1β and IL-6 both increased gradually on day 1 after modeling, significantly up to the A2 (IL-1β, *P* = 0.016; IL-6, *P* = 0.014), S2 (IL-1β, *P* = 0.000; IL-6, *P* = 0.000), and S3 groups (IL-1β, *P* = 0.000; IL-6, *P* = 0.000), and gradually recovered on day 7 after the treatment (Figure 3A-B). As shown in Figure 3C, TNF-α only significantly increased on the S2 (*P* = 0.005) and S3 (*P* = 0.015) groups. Thus, anesthesia and surgical stimulation could cause inflammatory cytokine expression in rats. The serum level of inflammatory cytokines after anesthesia was lower than that in the corresponding surgery group, indicating that inflammatory cytokine expression was more affected by surgery stimulation than anesthesia, consistent with our previous behavior test of POCD.

The effect of anesthesia and splenectomy on neuronal loss and damage in the CA1 area of hippocampus was determined by HE staining. In the control group, under the light microscope, we saw more neurons that were arranged orderly and had large and round nuclei (Figure 3D). In the S2 group, the number of neurons was reduced with irregular arrangement. Likewise, the S3 group had a disordered arrangement of neurons. Meanwhile, we utilized the Nissl staining to determine neuronal damage. In the control group, the neuron bodies were round or pyramidal and regularly arranged, and more Nissl bodies had larger nuclei (Figure 3E). However, the number of neurons was less, with irregular arrangement and less Nissl bodies in the S2 and S3 groups. Thus, surgery stimulation could cause hippocampal damage and further cognitive impairment.

### MR was activated in the hippocampus under cognitive impairment condition

**Figure 4:**
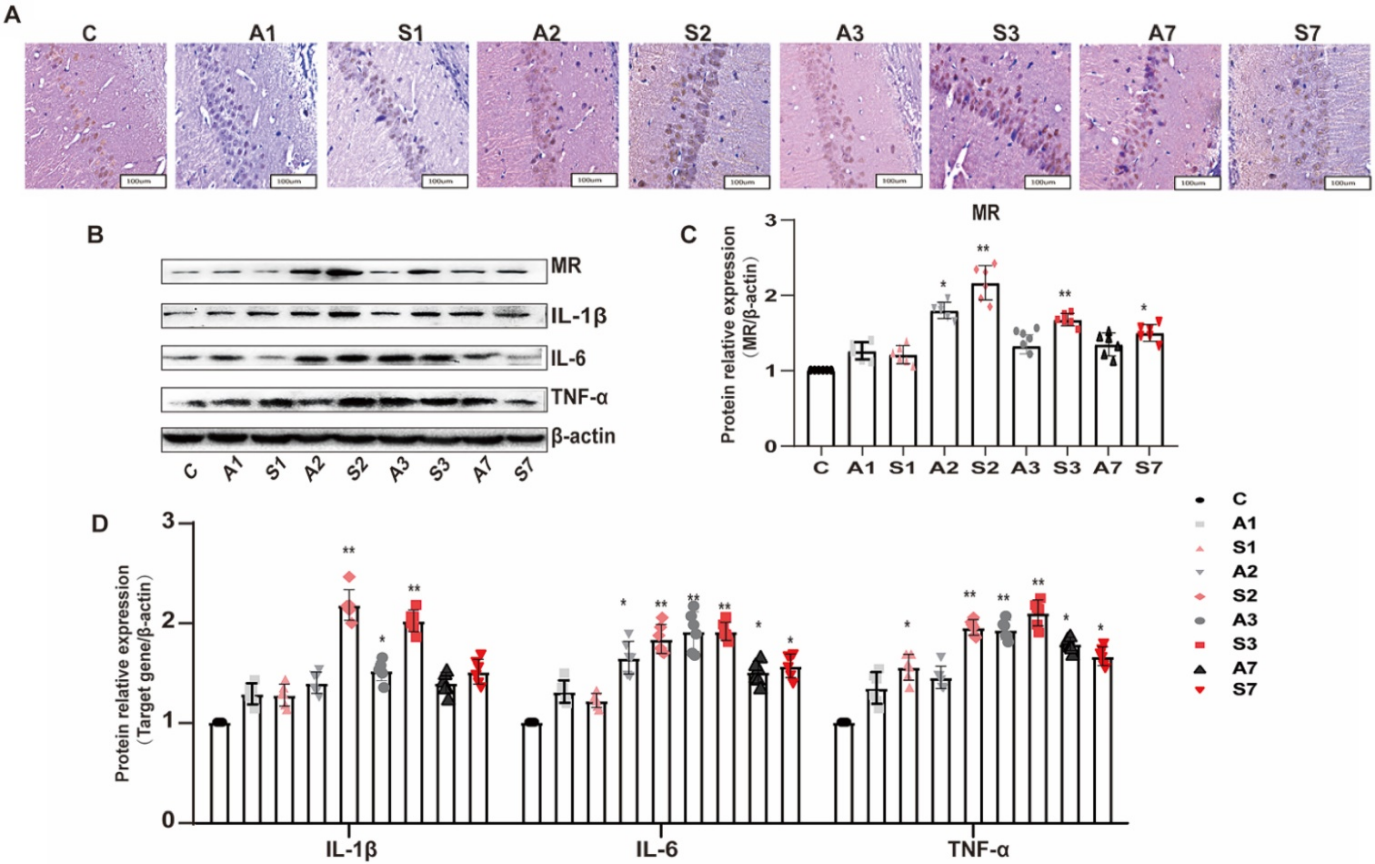
** Upregulation of MR in the hippocampus under cognitive impairment condition.** (A) The MR expression in the hippocampal CA1 region was detected by immunohistochemistry among the groups. (B-D) The protein expression of MR, IL-1β, IL-6, and TNF-α among the groups was assayed using Western blot. Data are presented as the mean ± SD. *P < 0.05 and **P < 0.01 vs. the control group. Control group, n = 6; A1, A2, A3, and A7 groups, n = 6; S1, S2, S3, and S7 groups, n = 6.

To further detect the protein expression of MR in the hippocampus, we applied immunohistochemistry to observe the MR expression in the CA1 region of the hippocampus. As shown in Figure 4A, the MR protein expression was positive in the CA1 area of the hippocampus across the groups. Compared with the control group, the MR expression in the S2 and S3 groups significantly increased; the other groups also showed an upward trend after treatment.

The expression of MR and inflammatory cytokines in the hippocampus of rats was further detected by Western blot to explore the relationship between MR and POCD. The expression of MR and the cytokines IL-1β, IL-6, and TNF-α in the hippocampus was increased after surgery stimulation (Figure 4B-D). The surgery group had a higher and longer inflammatory cytokine expression than the anesthesia group. The expression of MR and inflammatory cytokines was parallel to cognitive impairment; thus, the MR in the hippocampus might participate in the inflammatory response, leading to the increased expression of inflammatory cytokines in the hippocampus and peripheral serum, and cognitive impairment.

### EPL could improve cognitive dysfunction

**Figure 5:**
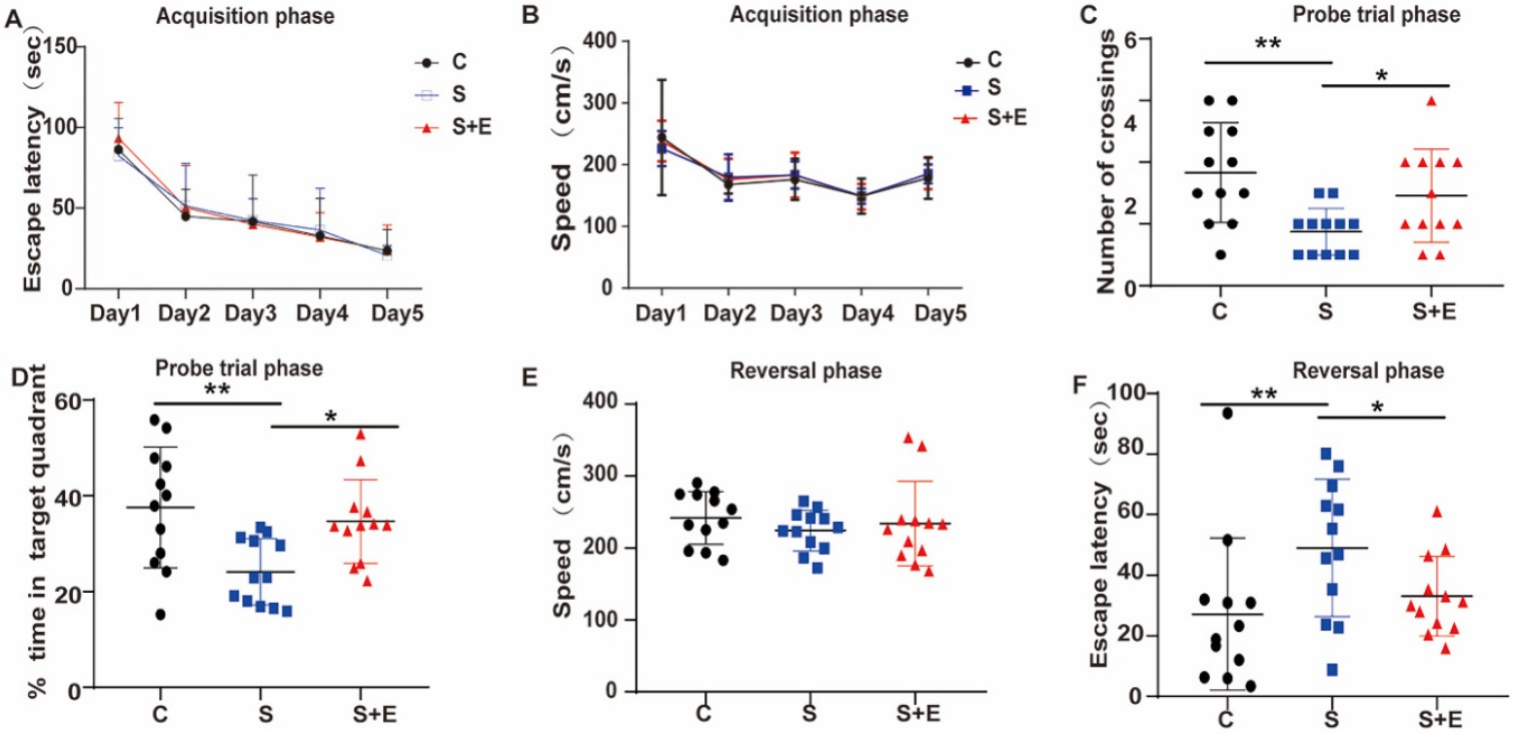
** MR blocker EPL could improve cognitive dysfunction.** (A) Escape latency during the acquisition phase for 5 training days. The probe trial and reversal phases were performed on day 2 after treatment. Frequency of crossing the platform (B) in the probe trial phase. (E) Escape latency and (F) speed in the reversal phase. Data are presented as the mean ± SD. *P < 0.05 and **P < 0.01 vs. control group. Control group (C, n = 12), Surgery and EPL treatment group (S+E, n = 12), Surgery group (S, treatment with same amount of normal saline, n = 12).

To further explore the role of MR in cognitive dysfunction, we examined the effect of the MR-specific blocker EPL on hippocampal neuron inflammation and POCD. To demonstrate whether EPL could improve cognitive dysfunction, we divided the rats into the Control, Surgery, and Surgery and EPL treatment groups. During the acquisition phase, the escape latency decreased in all groups (Figure 5A). Probe trial test and reversal test were conducted at day 2 after performing interventions based on MR expression in our experiments. Compared with the Control group, the Surgery group had lower frequency of crossing the platform, shorter time of staying in the quadrant, and longer escape latency (*P* < 0.01, Figure 5C, D, and F). In addition, the Surgery and EPL treatment group had shorter escape latency, higher frequency of crossings, and longer time in the quadrant than the Surgery group (*P* < 0.05, Figure 5C, D, and F). Meanwhile, the average swimming speed of each group was not significantly different (*P* > 0.05, Figure 5B and E). Therefore, EPL could improve cognitive impairment.

### Effect of EPL on inflammatory cytokines and hippocampal neurons

**Figure 6:**
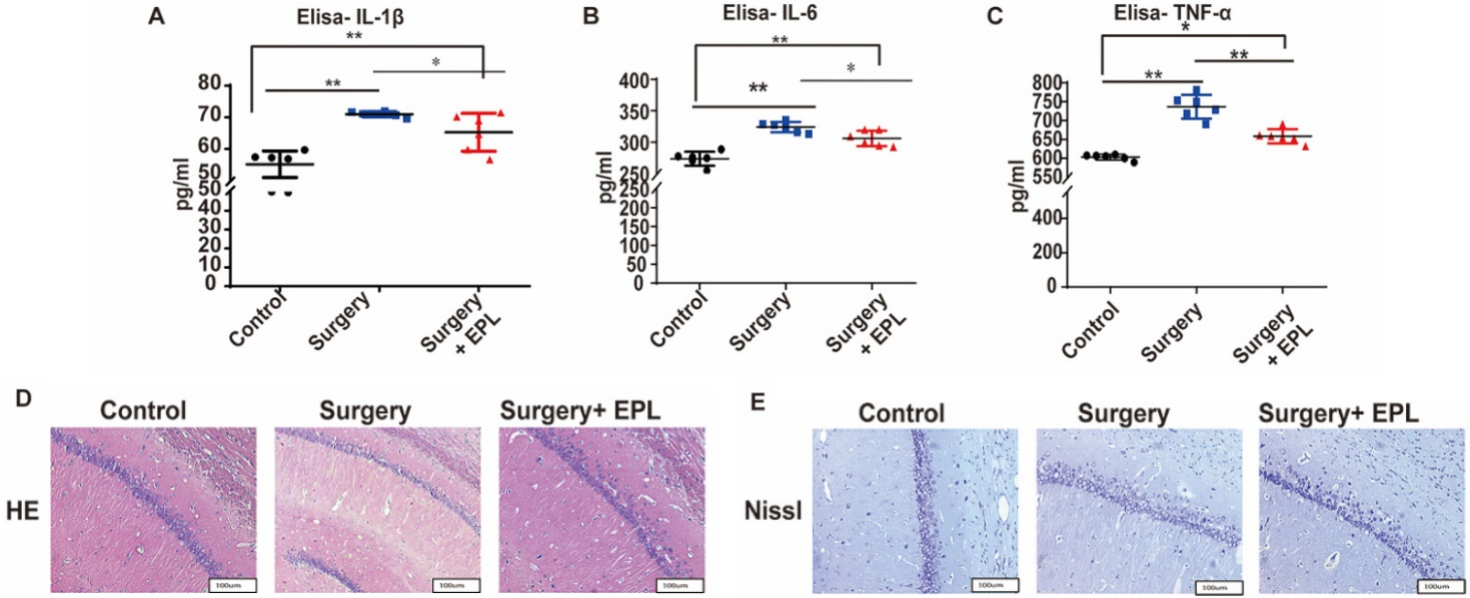
** Effect of EPL on inflammatory cytokines and hippocampal neurons.** The expression of the serum inflammatory cytokines such as (A) IL-1β, (B) IL-6, and (C) TNF-α among the groups was assayed using ELISA. (D) HE and (E) Nissl staining was applied in the hippocampal CA1 region to detect the histopathological changes of hippocampal neurons among the groups. Data are presented as the mean ± SD. *P < 0.05 and **P < 0.01 vs. the control group. Control group (C, n = 6), Surgery and EPL treatment group (S+E, n = 6); Surgery group (S, treatment with same amount of normal saline, n = 6). 3.6 EPL could protect the hippocampus by inhibiting MR protein overexpression.

According to previous results, MR is associated with neuronal inflammatory response in cognitive dysfunction. Therefore, we measured the serum IL-1β, IL-6, and TNF-α levels. The expression of IL-1β, IL-6, and TNF-α of the Surgery group and Surgery and EPL treatment group was significantly higher than those of the Control group (*P* < 0.01, Figure 6A-C). Meanwhile, the expression of IL-1β, IL-6, and TNF-α of the Surgery and EPL treatment group was significantly lower than those of the Surgery group (*P* < 0.05, Figure 6A-C). Therefore, EPL might improve the inflammatory reaction of rats with cognitive dysfunction.

As shown in Figure 6D, the HE staining revealed that the neurons in the Control group were orderly arranged, uniform in staining, and regular in cell shape, whereas those in the Surgery group were scattered, irregular, and uneven. In the EPL treatment group, the neurons were regular and orderly, with a clear structure and a slightly uniform staining, compared with the Surgery group. Meanwhile, the Nissl staining showed that the Nissl bodies of the control group were evenly distributed in the cytoplasm, with a clearly visible neuron structure (Figure 6E). In the Surgery group, the Nissl bodies were unclear, less, and unevenly stained. Similarly, the neurons in the Surgery and EPL treatment group were regular in cell shape and evenly stained, but more Nissl bodies were observed than those in the Surgery group. Thus, EPL treatment could improve hippocampal damage caused by surgery stimulation.

**Figure 7:**
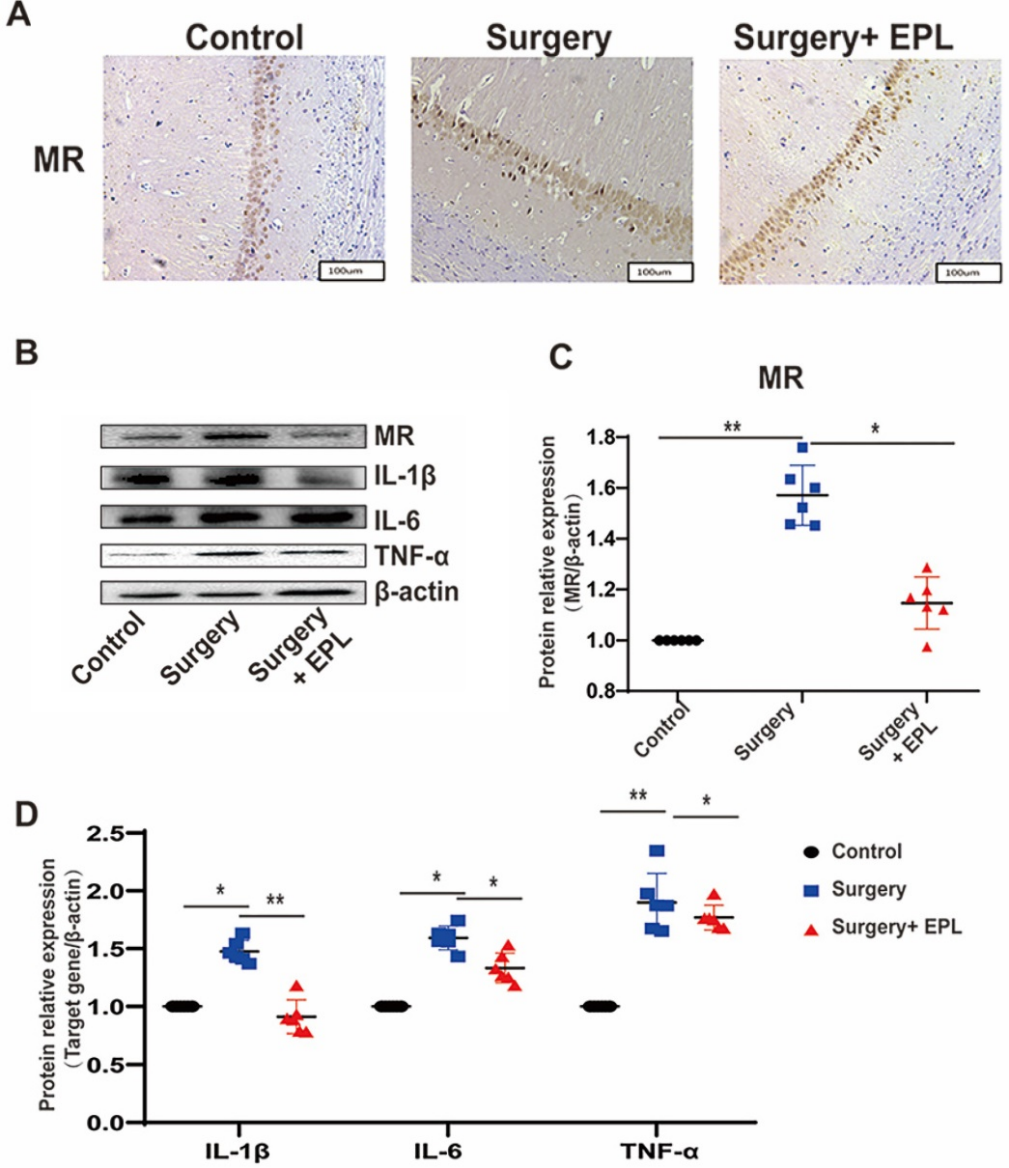
** EPL could protect the hippocampus by inhibiting MR protein overexpression.** (A) The MR expression in the hippocampal CA1 region was detected by immunohistochemistry among the groups. (B-D) The protein expression of MR, IL-1β, IL-6, and TNF-α among the groups was assayed using Western blot. Data are presented as the mean ± SD. *P < 0.05 and **P < 0.01 vs. the control group. Control group (C, n = 6), Surgery and EPL treatment group (S+E, n = 6), Surgery group (S, treatment with same amount of normal saline, n = 6).

We further detected whether EPL could protect the hippocampus by inhibiting MR protein overexpression. Immunohistochemistry results showed that the cytoplasmic staining of neurons in the control group was clear and even; additionally, the MR expression increased after surgery stimulation but was reversed after EPL treatment (Figure 7A). Western blot showed that the expression of MR and the cytokines IL-1β, IL-6, and TNF-α in the hippocampus was similar; it increased after surgery stimulation but was reversed after EPL treatment (Figure 7B-D). Overall, MR was upregulated in POCD, affecting the expression of inflammatory cytokines and causing hippocampal neuronal damage, but EPL inhibited MR protein overexpression, demonstrating a neuroprotective effect.

## Discussion

Neuroinflammation plays an important role in POCD pathophysiology. We hypothesized that upregulation of MR during anesthesia/surgery inflammation might increase POCD incidence. Consistent with our hypothesis, MR was upregulated in the hippocampus under cognitive impairment condition. In addition, oral administration of the MR-selective antagonist EPL could protect cognitive dysfunction by inhibiting the MR overexpression in the hippocampus.

**Figure 8:**
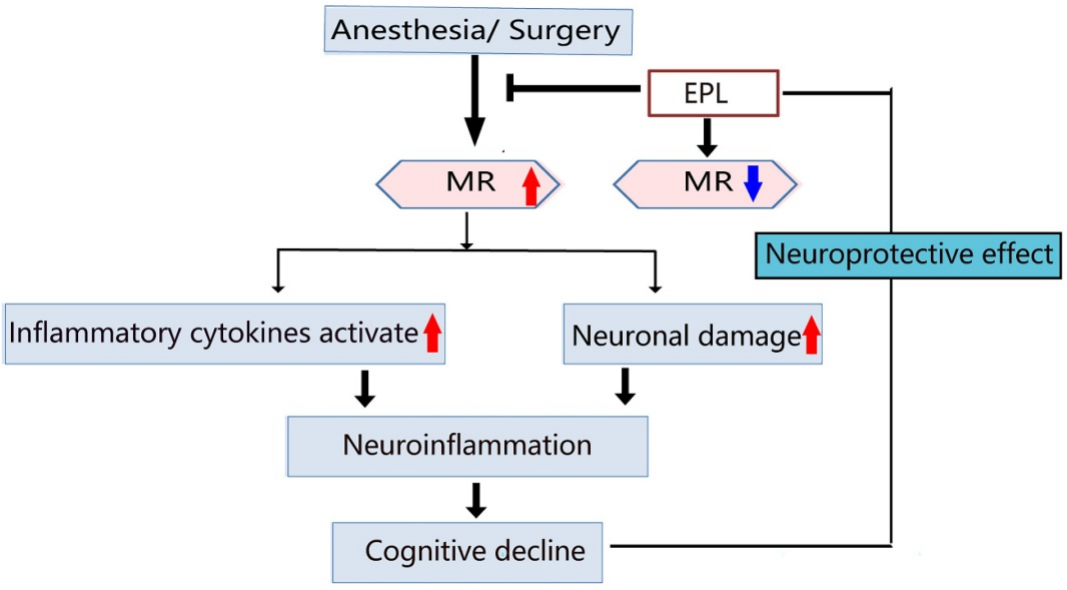
Schematic diagram for illustrating the MR and EPL treatment in cognitive decline induced by anesthesia and surgery.

POCD is a severe neurological sequela that may develop into mild cognitive impairment or even dementia 23, 24. Proinflammatory cytokines such as IL-1β and TNF-α play an important role in tissue damage caused by surgery, thereby activating systemic and CNS inflammation, ultimately leading to cognitive decline 9, 10. In addition, a large number of neurons in the frontal cortex and hippocampus is vital for maintaining normal learning and cognitive abilities 25, 26. The stress response caused by surgical trauma would activate the microglial function, release considerably numerous proinflammatory cytokines, and promote the formation of CNS inflammatory response, resulting in direct or indirect damage to neurons, and eventually trigger POCD. As shown in the summarized mechanisms in Figure 8, anesthesia and more evidently, surgery stimulation could lead to the increased expression of inflammatory cytokines and hippocampal neuronal damage, subsequently leading to neuroinflammation and cognitive impairment, which could be reversed by EPL treatment demonstrating a neuroprotective effect.

Anesthesia and more evidently, surgery stimulation could upregulate MR expression in the hippocampus, leading to the increased expression of inflammatory cytokines and hippocampal neuronal damage, subsequently causing neuroinflammation and cognitive decline, which could be reversed by EPL treatment as an MR-specific blocker through its neuroprotective effect.

MR Overexpression might aggravate the risk and severity of inflammatory reaction in diabetes and hypertension 27, 28. Notably, patients with POCD reportedly exhibit HPA axis activation and high cortisol levels 14, 15. Interestingly, the GR expression in the hippocampus is decreased in rats with cognitive impairment induced by material separation, which may amplify neuroinflammation and cause POCD 29. In our study, the MR expression in the hippocampus was increased, the proinflammatory cytokine expression was enhanced, and the neuroinflammation caused by surgical trauma was aggravated, eventually leading to cognitive impairment. The relationship between cortisol levels and cognitive function might originate from different roles of MR and GR that bind cortisol with evidently different affinity 30. Therefore, MR activation, GR inhibition, and HPA axis activation exerted vital roles for POCD development.

EPL as an MR-specific antagonist has been approved by the FDA for treating hypertension and heart failure 31. In our previous research 11, local EPL injection into the DRG could alleviate pain and decrease the excitability of small-diameter DRG neurons. MR overexpression impairs both TRPV4-mediated dilation of cerebral parenchymal arterioles and cognitive function, which could be improved by EPL treatment 32. Importantly, EPL might be a potential therapeutic approach to improve cerebrovascular function and cognition in patients with hypertension. Consistently, our results showed that EPL could improve the cognitive function of the POCD animal model by inhibiting the neuronal inflammatory response. Interestingly, EPL might be a better choice for patients with hypertension to undergo surgery because of its neuroprotective effects. Besides, microglial activation is an important response to neuroinflammation 33. Studies have shown that the number of necrotic neurons in hippocampal CA1 area decreases after EPL treatment, which is consistent with the decrease of proinflammatory cytokines in the whole body and hippocampus and the recovery of cognitive function. In addition, recent study has shown that activated microglia secrete inflammatory factors to promote the formation of A1 astrocytes 34. At the same time, a large number of A1 astrocytes have been observed preferentially in the lesion area in brain tissue samples from neurodegenerative diseases such as Alzheimer's disease, suggesting that A1 formation may drive neurodegeneration in the disease. It is necessary to further study the exact mechanism of microglial activation induced by splenectomy or EPL.

Meanwhile, limitations and deficiencies should be considered in our study. First, we did not systematically study the effect of surgical trauma on the function of HPA axis but focused on hippocampal MR expression, which is closely related to HPA axis function 35. Furthermore, EPL was orally administered preoperatively in our study. In view of the extensive activity of MR expression in tissues, the additional intraventricular injection of MR shRNA might determine the effect of MR overexpression in POCD development. Moreover, the elevation of inflammatory cytokines was probably a direct effect of surgical trauma or an indirect effect by surgical trauma-MR-inflammation circle. The relationship between MR and inflammation in POCD needs further investigation.

There are several caveats that must be considered. Although splenectomy has been selected as the model of peripheral surgery in many studies of POCD 36-38, the spleen is an important lymphoid organ related to innate and adaptive immune response. Splenectomy might interfere with immune results, but not with surgical trauma. Splenectomy itself can change the inflammatory response of organisms. However, this leads to reduced inflammation rather than proinflammatory status. At the same time, since MR is produced by the kidney, it is necessary to consider the impact of other operations such as nephrectomy on this study. Therefore, further studies, including the evaluation of other peripheral surgical methods, will better determine the role of MR in the neuropathogenesis of POCD. In addition, a series of studies have demonstrated the contribution of advanced age as risk factor exerts to POCD 36-38. Remarkably, the cognitive dysfunction in our study did not persist for 7 days after surgery; therefore, the phenomenon we observed may better reflect the cognitive impairment associated with delirium. Delirium is a postoperative state that does not necessarily develop into POCD, which may require additional stimulation (such as infection, advanced age, or potential neurological disease) to translate this phenomenon into more persistent cognitive impairment. Therefore, the elderly rats might be a better choice in our further study of POCD model to show more obvious effect.

In conclusion, this preclinical study indicated that MR may play an important role in POCD involving neuroinflammation. Some MR effects may be activated in the level of hippocampus under cognitive impairment condition. However, the MR-specific blocker EPL could alleviate the inflammatory activation and neuronal damage by exerting neuroprotective effects. Overall, our research implied that MR was overexpressed via neuroinflammation regulation in the brain in splenectomy induced cognitive decline and EPL is a promising drug for POCD.
